# Influence of Gaseous Ozone Treatments on Mechanical and Chemical Properties of Japanese Quince Fruits During Storage

**DOI:** 10.3390/foods14193412

**Published:** 2025-10-03

**Authors:** Oskar Basara, Miłosz Zardzewiały, Piotr Kuźniar, Stanisław Pluta, Justyna Belcar, Józef Gorzelany

**Affiliations:** 1 Department of Food and Agriculture Production Engineering, University of Rzeszow, 4 Zelwerowicza Street, 35-601 Rzeszów, Poland; 2 Department of Horticultural Crop Breeding, National Institute of Horticultural Research (InHort), Konstytucji 3 Maja 1/3 St., 96-100 Skierniewice, Poland; 3 Farming Cooperative SAN, Łąka 598, 36-004 Łąka, Poland

**Keywords:** *Chaenomeles japonica*, gaseous ozone, cold storage, mechanical properties, chemical composition

## Abstract

*Chaenomeles japonica* (*Chaenomeles japonica* Thunb. Lindl. ex Spach.) is gaining increasing attention due to its high nutritional value and potential for industrial use. The development of new breeding clones (potential new cultivars) with improved morphological and chemical properties is essential for enhancing its commercial cultivation. In this study, the impact of ozone in its gaseous form and cold storage on the morphological and chemical properties of newly selected Polish clones of *Chaenomeles japonica* was determined. Breeding clone ‘3b/1’ produced the largest fruits, with a significantly higher average weight of 99.8 g compared to other clones. Fruits of clones ‘3b/1’ and ‘7d/8’ had the greatest tolerance to mechanical damage, requiring the highest force and energy for puncture and showing the most extensive deformation. The highest ascorbic acid content was recorded in clone ‘4c/1’ (117.3 mg·100 g^−1^), while clone ‘3b/1’ had the highest total phenolic content, reaching 373.92 mg GAE·100 g^−1^. A 15 min ozone treatment led to an average increase of 5.3% in both ascorbic acid and total phenolic content. In contrast, cold storage for 60 days caused a reduction of approximately 29.66% of ascorbic acid. Clone ‘3b/1’ appears to be the potential new Polish cultivar and an introduction for cultivation due to its large fruit size, their high mechanical tolerance and relatively favorable chemical composition.

## 1. Introduction

*Chaenomeles japonica* Thunb. Lindl. ex Spach., commonly referred to as Japanese quince (JQ), is a shrub native to East Asia and has been used in traditional Chinese medicine for over three millennia. Belonging to the *Chaenomeles* genus—one of the oldest cultivated groups within the Rosaceae family—this species is taxonomically closely related to the genera *Cydonia*, *Pyrus* and *Malus* [1]. Fruits of *Chaenomeles japonica* consistently emerge as rich sources of ascorbic acid, chlorogenic acid, epicatechins, diverse flavonoids and triterpenes (notably in leaves), alongside seed oil constituents such as unsaturated fatty acids, tocopherols, phytosterols and carotenoids. These compounds underline its potential as both a functional food ingredient and a phytopharmaceutical resource [2,3,4]. *Chaenomeles japonica* fruits provide a wide spectrum of pharmacological activities, including anti-inflammatory, anticancer, analgesic, antioxidant, hepatoprotective, neuroprotective, antibacterial and antiviral effects. It plays a critical role in the stabilization and biosynthesis of collagen fibers, serving as a key contributor to the conversion of proline into hydroxyproline—an essential step in collagen formation [5]. Selecting the right cultivars is crucial when establishing commercial plantations. It significantly impacts storage capacity and reduces mechanical damage. *Chaenomeles japonica* fruit has a shelf life exceeding three months, and cold storage has been shown to significantly reduce postharvest losses [6,7]. However, delayed harvesting and prolonged cold storage are associated with a decline in fruit firmness and an increase in surface browning. One of the major postharvest challenges in the commercial handling of *Chaenomeles japonica* cultivars is enzymatic browning, a physiological disorder that negatively affects fruit quality [8]. This disorder is influenced by a combination of pre-harvest factors, harvest timing and storage conditions. Mechanical harvesting and postharvest storage of fruit not only lead to a loss of turgor due to water loss but also induce significant alterations in the fruit’s chemical composition. Pre-processing operations such as cleaning and rinsing can negatively impact on the quality of raw fruit material, contributing to increased water loss and inducing changes in texture and firmness [9,10].

Ozonation is a non-thermal and environmentally sustainable method of food preservation that maintains the sensory and nutritional quality of fruit. The application of ozone gas alters the phenolic compound profile and their content [11]. Application of O_3_ at suitable concentrations may regulate metabolic pathways, resulting in higher levels of bioactive compounds and improved antioxidant potential [12,13]. Gaseous ozonation has a beneficial effect on post-harvest fruit storage by reducing water loss and microbial contamination, as well as suppressing ethylene emission, thereby extending the shelf life of the treated produce and fruits. In the fruit and vegetable industry, ozone is valued for its disinfecting and biocidal effects, as it decomposes rapidly into oxygen and does not produce harmful residues [14,15]. The use of gaseous ozone, due to its effect on morphology and the content of chemical compounds, can be used in the production of *Chaenomeles japonica*. Ozone treatments may improve the content of chemical compounds and the fruit’s tolerance to mechanical damage.

The aim of our studies was to evaluate the effects of ozone treatment on selected mechanical and chemical properties of newly developed breeding clones of *Chaenomeles japonica (Chaenomeles japonica)* during storage. Fruits obtained from the field cultivation were analyzed for key bioactive compounds, including total polyphenol content, vitamin C levels and antioxidant capacity. During the storage stage, the fruit’s morphological properties and tolerance to mechanical damage were measured.

## 2. Materials and Methods

### 2.1. Materials

The study material consisted of fruits from seven new Polish breeding clones of *Chaenomeles japonica*: 3b/1, 4c/1, 7c/10, 7d/8, 8c/12, 10d/8 and 12b/5.

Clones are the result of the applied breeding program and selection work conducted at the Department of Horticultural Crop Breeding of the National Institute of Horticultural Research (InHort) in Skierniewice, central Poland. Fruits were manually harvested in 2024 from bushes of these genotypes cultivated in a field trial at the Pomological Orchard of the InHort in Skierniewice (51°57′38″ N, 20°08′39″ E, Łódzkie Voivodeship, Poland). The fruits were harvested at full ripeness, determined based on skin coloration, their typical aroma and brown or dark brown seeds inside fruits (after their cutting). The fruits were stored for a period of 3 months, in plastic containers located in a refrigerator at 4 °C and 80% relative humidity. During fruit storage, selected chemical and mechanical properties were systematically evaluated. Analysis was performed three times.

### 2.2. Ozone Treatment

Fruits representing all evaluated genotypes were randomly distributed into three groups, 2000 g per group. The first group acted as the control and remained untreated, whereas the other two groups underwent ozonation in plastic containers measuring 0.6 × 0.4 × 0.4 m (L × W × H). Gaseous ozone was applied at a concentration of 10 ppm for durations of 15 and 30 min, with a flow rate of 40 g O_3_·h^−1^. Ozone was generated using KORONA A 40 Standard (Korona, Piotrków Trybunalski, Poland), and concentration of gaseous ozone was measured using a 106 M UV Ozone Solution Detector (Ozone Solution, Hull, MA, USA). The ozonation process of Japanese quince fruit was carried out under strictly controlled conditions at a temperature of 20 ± 1 °C.

### 2.3. Determination of Water Content

Determination of water content in *Chaenomeles japonica* fruits was carried out by the drying method (105 °C) according to the PN-90/A-75101-03:1990 [16] in a laboratory dryer SLW 115 SMART (POL-EKO, Wodzisław Śląski, Poland). Samples of fruits were dried after 1 day of storage post-harvest, and again after 30 and 60 days of cold storage, with random selection for testing. The data are expressed in percentages (%), and each set of measurements was repeated three times.

### 2.4. Evaluation of Morphological Features of Chaenomeles japonica Fruits

The morphological properties of the fruits of the tested clones were examined immediately before the mechanical properties were measured. Length and diameter were determined to the nearest 0.01 mm, whereas weight was measured to the nearest 0.001 g.

Subsequently, the sphericity coefficient (*φ*, %) was calculated using the following equation:

(1) $$\varphi =\frac{{(LD}^{2})\frac{1}{3}}{L}100\%,$$

where

*φ*—sphericity [%];
*L*—length of the fruit [mm];
*D*—diameter of the fruit [mm].

The density (kg·m^3^) of individual fruits was determined by dividing their mass by the volume, where volume was approximated using the formula for an ellipsoid based on length (*L*) and diameter (*D*) [17].

### 2.5. Determination of Fruits Color

The analysis of the fruits color was conducted via a reflection method by using a colorimeter CP100 (3Color, Narama, Polska). Color measurements were conducted using the CIE Lab colorimetric system [18], which records L (lightness), a* (red–green) and b* (yellow–blue). Ten measurements were taken for each fruit sample. The reflectance method was applied under standard illumination and a 2° standard observer.

### 2.6. Mechanical Properties of Chaenomeles japonica Fruits

Mechanical parameters of *Chaenomeles japonica* were assessed through compression testing between two horizontal plates using a Brookfield CT3-1000 texture analyzer (AMETEK Brookfield, Middleboro, MA, USA) controlled by TexturePro CT software 1.2. The assay was performed under a pre-tension of 0.1 N, with the pressing plate moving at 0.5 mm·s^−1^. For each measurement, the maximum breaking force F (N), rupture energy (mJ), deformation λ (mm) and fruit diameter d (mm) were determined and subsequently expressed as percentages.

### 2.7. Determination of pH and Acidity

Total acidity, expressed as citric acid, and pH of *Chaenomeles japonica* fruits were determined by potentiometric titration with 0.1 M NaOH to an endpoint of pH 8.1 using a TitroLine 5000 analyzer (SI Analytics, Mainz, Germany), following the PN-EN12147:2000 standard [19]. All analyses were conducted in triplicate.

### 2.8. Determination of Bioactive Components

The contents of ascorbic acid in *Chaenomeles japonica* fruits were measured in accordance with PN-A 04019:1998 [20]. The results were expressed in mg GAE·100 g^−1^, GAE—Gallic Acid Equivalents. The total polyphenol content (TPC) was determined using the Folin–Ciocalteu method according to the methodology of Belcar et al., 2022 [21]. The results were expressed in mg·100 g^−1^. The free radical scavenging activity (DPPH method) was determined using the method described in Česonienė et al., 2021 [22] and expressed as mM TE·100 g^−1^. Antioxidant activity was evaluated using the ABTS assay according to Raudonė et al. [23], with results expressed as µM TE·100 g^−1^, TE—Trolox equivalents. The ferric-reducing antioxidant power (FRAP) was determined following Orsavová et al. [24] and expressed as mM Fe^2+^·100 g^−1^. Each analysis was carried out in triplicate.

### 2.9. Statistical Analysis

Statistical analysis of the collected data was performed using Statistica 13.3 software (TIBCO Software Inc., Tulsa, OK, USA). The analysis included analysis of variance (ANOVA) and the Least Significant Difference (LSD) test at a significance level of α = 0.05.

## 3. Results and Discussion

### 3.1. Determination of Morphological Features of Chaenomeles japonica

Long-term storage in refrigerated conditions can cause unfavorable morphological and chemical changes in fruit. Increased fruit respiration causes water loss, which affects fruit weight [25]. The morphological properties of fruit during storage varied depending on the clone and storage period (Table 1).

Analysis of variance showed a significant effect of clone on all morphological characteristics of fruit and storage time on their length, diameter and weight. The mean values of fruit length, width and weight were similar to the results of Smatova et al. [26] and Stalažs et al. [27], who reported an average width of approximately 40 mm and a weight of approximately 50 g. The largest fruits were obtained for clone 3b/1, while the smallest for 7c/10. A high sphericity index was recorded for clone 4c/1, and the highest density for 10d/8. Storage for 30 and 60 days resulted in a significant reduction in fruit dimensions and weight—after 2 months, the weight was over 20% lower compared to fresh fruit. The results indicate that storage longer than 30 days negatively affects the production value and attractiveness of fruit. Ozonation can significantly reduce weight loss. Ozonated fruit reduces gas exchange, resulting in increased water retention within the fruit. This can significantly increase fruit firmness, thus improving its commercial quality [28,29].

### 3.2. Determination of Mechanical Propeties

Mechanical fruit harvesting, transport and storage of fruits may have a negative impact on the mechanical properties of the fruits, thus reducing their further storage capacity and biological value [30]. The water content and mechanical properties of the tested *Chaenomeles japonica* clones varied depending on the tested genotype and storage time (Table 2).

A significant effect of clone type and storage time on mechanical properties and water content was observed, as well as the effect of ozonation on fruit deformation and destruction energy. The average water content (85.50%) was consistent with previous results by Tarko et al. [31], who reported a dry matter value of 12.9%. Fruits of clones 3b/1 and 7d/8 were the most resistant to damage, while 8c/12 and 10d/8 were the least resistant, with the latter having the highest water content. Fruits of clone 7c/10 contained the least water. It was noted that unozonated fruits exhibited the greatest deformation, while ozonated for 15 min showed increased deformability. During storage, water content decreased significantly, while strength, energy and degree of deformation increased; after 60 days, deformation was over 40% greater than in fresh fruit.

In the study of Cheng et al. [32], storing strawberry fruits in an ozonated atmosphere increases the firmness of the fruit. Similar relationships were described by Juhnevica-Radenkova et al. [33], indicating an increase in the force required to puncture the fruit during storage. The results suggest that longer storage reduces the functional and potentially chemical value of the fruit. Immersion of grapefruit in ozonated water (0.6 ppm, 5 °C) reduced the weight loss (physiological weight loss) and after 40 days of storage resulted in a higher water content in these fruits compared to the control sample [34]. Zapałowska et al. [35] found that the cyclical use of gaseous ozone in the storage *of Hippophae rhamnoides* L. fruits increased their resistance to mechanical damage, after 7 days of storage, fruits exposed to gaseous ozone had significantly lower water loss (reduced water loss) compared to the control. Moreover Matłok et al. [36], cyclically applied ozonation (gaseous ozone, e.g., 10 ppm for 15 min per day) significantly reduced water loss (lower water loss) compared to the control fruits.

### 3.3. Determination of Fruits Color

Fruit storage and processing procedures can affect their morphological properties, including fruit color. Color is one of the most important quality characteristics of fruit; consumers primarily consider its appearance when selecting fruit for buying [37]. The tested *Chaenomeles japonica* fruits showed color differences influenced by clone, ozonation time and storage conditions (Table 3).

Analysis of variance revealed a significant effect of tested clones and storage time on all color space coordinates determined for them, as well as ozonation time on lightness (L*) and redness (a*). The results are comparable to those obtained in the study of Rubinskienė et al. [38] and Turkiewicz et al. [39], where the fruits tested in the experiment were characterized by comparable L*, a* and b* values. The average color values in the fruits were as follows: L*—65.95, a*—13.77, b*—53.54. The lowest lightness (L*), yellowness (b*) and the highest redness (a*) were observed in the clone 7d/8. The highest lightness (L*) and the lowest redness (a*) were observed in clone 3b/1, and the highest yellowness (b*) in clone 8c/12. Ozonation of *Chaenomeles japonica* fruit only reduced their lightness. However, during storage, the lightness and yellowness decreased, while the redness increased. Damage that may occur during harvesting and long-term storage may negatively affect the color of the fruit, which may significantly reduce the commercial value of the fruit [40].

### 3.4. Chemical Properties of Chaenomeles japonica Fruits

*Chaenomeles japonica* is known for the yellow fruits characterized by a chemical composition rich in numerous health-promoting substances, including phenolics, organic acids, terpenoids, alcohols, ketones and aldehydes [41]. These fruits are also known for their high vitamin C content and potential therapeutic benefit. The fruit has shown hepatoprotective, anti-inflammatory, antioxidant, neuroprotective and antimicrobial properties [42,43]. The content of chemical compounds in fruits influences not only the health-promoting value of fruits but also their taste and sensory experience [44]. In the analyzed *Chaenomeles japonica* clones, pH and total acidity values changed as a function of genotype, storage time and ozone exposure (Table 4).

Analysis of variance revealed a significant effect of clone and ozone exposure time on titratable acidity values. The pH varied between tested clones and different doses of ozone. The average pH and total acidity values in the fruits of breeding clones were 2.99 and 3.58, respectively. The results of the above-mentioned values are comparable to those obtained in the study of Helin et al. [45] and Marat et al. [3]: the average pH value was 2.8 and the total acidity was 3.5% in the fruit samples of *Chaenomeles japonica*. The levels of bioactive compounds in fruits of the examined *Chaenomeles japonica* clones differed according to clone, storage duration and ozonation time (Table 5).

Analysis of variance revealed a significant effect of clone, time of ozone exposure and storage time on titratable acidity values. The ascorbic acid content in the tested *Chaenomeles japonica* clones ranged from 105.7 to 117.3 mg·100 g^−^^1^. Breeding clone ‘4c/1’ was characterized by the highest vitamin C content among the tested genotypes. The findings of this study are consistent with those reported in studies by Rubinskienė et al. [38] and Baranowska-Bosiacka et al. [46]. These authors presented that the vitamin C content in *Chaenomeles japonica* fruit varied between 72.00 and 127.50 mg·100 g^−^^1^. A 15 min ozone treatment was found to significantly raise the ascorbic acid concentration in the fruits of the analyzed clones by 5.3%. However, the longer the fruits were stored, the lower ascorbic acid content was analyzed; the fruit stored for 60 days had a 29.66% lower ascorbic acid content. The chemical composition of fruits can alter during extended storage periods, and improper storage conditions may result in a reduction in health-beneficial compounds, such as vitamin C [47].

A significant effect of clones, time of ozone exposure and storage time on the total phenolic content (TPC) were found. The average value of TPC was 360.52 mg GAE·100 g^−^^1^, and the clone ‘3b/1’ was characterized by the highest TPC—373.92 mg GAE·100 g^−^^1^. The average phenolic compound content in fruits obtained in this study was within the scope with values ranging from 185 to 413 mg GAE·100 g^−^^1^, reported by Ros et al. [48]. As in the case of the analysis of ascorbic acid content, a similar trend can be observed for the influence of ozonation and storage. The use of ozone gas for 15 min increased the TPC by 5.3%, and storage for 60 days reduced the TPC by 17.9%. However, the use of ozone gas treatment in the appropriate dose can significantly influence the content of bioactive compounds in fruits, increasing their health-promoting properties [49].

The antioxidant properties of the tested *Chaenomeles japonica* clones varied depending on the genotype, storage time and ozonation, as well as the method of determination. The average antioxidant values of fruits of the tested *Chaenomeles japonica* clones are as follows: DPPH 2.35 mM TE·100 g^−^^1^, ABTS 1.61 mM TE·100 g^−^^1^ and FRAP 0.32 mM Fe·100 g^−^^1^. The results obtained in our studies for the tested breeding clones are not fully consistent with those reported by Radenkovs et al. [50] and Klymenko et al. [51]. These researchers determined the following average values for antioxidant methods: DPPH 3.7 mM TE·100 g^−1^ and ABTS 5.05 mM TE·100 g^−1^, but those results concerned the fruit analysis of different *Chaenomeles japonica* genotypes. The effect of ozonation varied depending on the treatment time; 15 min of ozonation increased DPPH and ABTS values. Fruit storage for 60 days resulted in decrease in antioxidant value, regardless of the method used for analyses. Storage for 60 days resulted in a decrease in the DPPH, ABTS and FRAP values by −12.5%, 11.5% and 14.2%, respectively, compared to the fresh fruits of *Chaenomeles japonica*. The lower TPC caused by long-term storage may have a negative impact on the reduction in antioxidant values in fruits [52].

Ozone acts as an abiotic elicitor—the plant perceives it as stress. This leads to increased synthesis of secondary metabolites (phenols, flavonoids, anthocyanins), which increase antioxidant capacity over time. During storage, enzymatic and non-enzymatic processes (e.g., polyphenoloxidase activity) occur that can either lower or increase FRAP. Piechowiak et al. [53] demonstrated that the enzymatic system responsible for generating small-molecule antioxidants directly involved in mitigating the effects of oxidative stress induced by ozone exposure is activated in fruit stored in an ozone atmosphere.

In the experiment by Balawejder et al. [54], ozonation of apple fruits at a dose of 1 ppm for 30 min significantly increased the content of phenolic compounds and the antioxidant value of ABTS. A similar reaction is observed in the experiment of Basara et al. [55], the use of gaseous ozonation at a dose of 5 ppm for 3 and 5 min resulted in an increase in the content of bioactive compounds. Based on the experimental results, it may find practical applications in the production and storage of *Chaenomeles japonica* fruit. Application of a 5 ppm dose for 15 and 30 min increased the content of health-promoting compounds in the fruit.

The phenomenon of a decrease in vitamin C concentration during fruit storage is well documented in the literature and is mainly due to the degradation of ascorbic acid under the influence of factors such as oxygen, light and temperature [56,57]. According to Zheng et al. [58], vitamin C is one of the nutrients most sensitive to oxidation, and its stability during storage depends largely on storage conditions, including temperature and air access.

## 4. Conclusions

The results of this study indicated significant differences in the morphological and chemical characteristics of the newly tested *Chaenomeles japonica* clones. A significant effect of gaseous ozone and long-term storage on the above-mentioned features was also determined. The fruits of clone ‘3b/1’ were characterized by the largest fruits with a significantly higher weight of 99.8 g, compared to the rest of the tested clones. The fruits of clones ‘3b/1’ and ‘7d/8’ showed the highest tolerance to mechanical damage, as they needed considerably more force and energy to puncture and experienced the most deformation. The highest content of ascorbic acid was found in clone 4c/1: 117.3 mg·100 g^−1^, and the highest TPC was found in clone 3b/1: 373.92 mg GAE·100 g^−1^. Ozone gas used for 15 min resulted in an average 5.3% increase in ascorbic acid and TPC. Storage for 60 days resulted in an average 29.66% decrease in vitamin C and TPC. The use of the clone ‘3b/1’ may be the greatest application due to the large size of the fruit, high tolerance to mechanical damage and relatively good chemical composition.

## Figures and Tables

**Table 1:** Effects of genotypes and storage duration on morphological properties of *Chaenomeles japonica* fruits.

Variables		Length (mm)	Diameter (mm)	Sphericity (%)	Weight (g)	Density (10^−3^ kg·m^−3^)
Breeding clone	3b/1	57.49 ± 4.58 e	56.43 ± 4.97 e	98.68 ± 3.14 cd	99.85 ± 21.97 f	1.035 ± 0.078 b
	4c/1	48.25 ± 5.13 d	52.45 ± 5.68 d	105.20 ± 4.22 f	68.60 ± 18.60 e	0.988 ± 0.090 a
	7c/10	39.02 ± 2.28 a	40.32 ± 2.00 a	102.19 ± 2.96 e	37.41 ± 5.70 a	1.123 ± 0.062 d
	7d/8	45.59 ± 3.62 bc	45.57 ± 3.25 c	100.01 ± 3.16 d	53.16 ± 12.07 d	1.059 ± 0.073 bc
	8c/12	46.81 ± 3.49 cd	44.43 ± 3.38 bc	96.42 ± 2.77 bc	51.44 ± 10.05 cd	1.063 ± 0.099 c
	10d/8	47.02 ± 4.41 cd	41.57 ± 3.98 ab	91.90 ± 3.71 a	49.76 ± 13.93 bc	1.159 ± 0.049 d
	12b/5	44.52 ± 3.26 b	42.81 ± 3.29 b	97.39 ± 3.38 b	44.25 ± 12.15 ab	1.018 ± 0.073 ab
Duration of storage	1st day	49.45 ± 7.06 b	48.43 ± 7.94 b	98.37 ± 4.61 a	67.45 ± 29.06 b	1.065 ± 0.086 a
	30th day	45.68 ± 6.40 a	45.34 ± 6.26 a	99.46 ± 5.86 a	53.60 ± 21.64 a	1.062 ± 0.104 a
	60th day	45.75 ± 4.99 a	44.91 ± 5.47 a	98.65 ± 4.94 a	52.29 ± 16.99 a	1.063 ± 0.090 a
Mean		46.96 ± 6.42	46.23 ± 6.78	98.83 ± 5.16	57.78 ± 23.99	1.064 ± 0.093

The data are expressed as mean values (n = 10) ± SD. SD—standard deviation. Mean values within columns with different letters are significantly different (*p* < 0.05).

**Table 2:** Influence of breeding clone, storage length and gaseous ozonation on moisture content and mechanical properties of *Chaenomeles japonica* fruits.

Variables		Moisture Content (%)	Force (N)	Deformation (mm)	Energy (mJ)
Breeding clone	3b/1	85.28 ± 1.21 b	38.63 ± 6.13 d	3.34 ± 1.50 d	87.08 ± 21.88 c
	4c/1	85.61 ± 0.40 b	35.77 ± 4.40 c	2.29 ± 0.91 bc	78.99 ± 14.29 b
	7c/10	83.45 ± 1.55 a	36.24 ± 4.24 c	2.41 ± 0.88 c	77.73 ± 11.94 b
	7d/8	85.88 ± 0.96 b	39.37 ± 7.61 d	2.56 ± 1.10 c	81.83 ± 18.55 b
	8c/12	85.45 ± 1.22 b	28.64 ± 4.92 a	2.52 ± 1.11 c	61.73 ± 13.11 a
	10d/8	87.78 ± 0.63 c	30.43 ± 4.66 b	1.77 ± 0.75 a	56.84 ± 10.15 a
	12b/5	85.00 ± 1.12 b	36.81 ± 5.13 c	1.99 ± 0.54 ab	80.41 ± 14.05 b
Ozone exposure time	0 min	85.45 ± 1.63 a	35.35 ± 6.87 a	2.36 ± 1.03 a	76.36 ± 18.86 b
	15 min	85.53 ± 1.65 a	34.97 ± 6.45 a	2.53 ± 1.19 b	73.78 ± 17.36 a
	30 min	85.50 ± 1.52 a	35.07 ± 6.40 a	2.34 ± 1.09 a	74.69 ± 19.01 a
Duration of storage	1st day30th day60th day	86.32 ± 1.08 c	33.73 ± 7.19 a	1.81 ± 0.36 a	69.05 ± 9.01 a
		85.71 ± 1.10 b	36.12 ± 6.58 b	2.50 ± 0.80 b	81.79 ± 14.39 b
		84.45 ± 1.86 a	35.53 ± 5.64 b	3.24 ± 1.16 c	83.99 ± 18.71 b
	Average	85.49 ± 1.59	35.13 ± 6.56	2.51 ± 1.11	78.27 ± 18.42

The data are expressed as mean values (n = 10) ± SD; SD—standard deviation. Mean values within columns with different letters are significantly different (*p* < 0.05). N—newton, mJ—millijoule.

**Table 3:** Color properties of *Chaenomeles japonica* fruits depending on the cultivar, duration of storage and time gaseous ozonation.

Variables		L*	a*	b*
Breeding clone	3b/1	70.91 ± 2.46 e	10.44 ± 2.12 a	57.00 ± 2.37 cd
	4c/1	69.32 ± 3.50 d	10.51 ± 2.17 a	55.69 ± 3.27 c
	7c/10	62.40 ± 5.24 b	18.35 ± 1.76 c	53.60 ± 7.35 b
	7d/8	58.71 ± 4.36 a	19.21 ± 1.57 d	48.60 ± 6.41 a
	8c/12	68.63 ± 2.01 d	13.37 ± 1.65 b	57.87 ± 2.00 d
	10d/8	65.87 ± 3.01 c	10.77 ± 2.47 a	49.89 ± 2.35 a
	12b/5	65.77 ± 2.54 c	13.75 ± 1.34 a	52.23 ± 2.34 b
Ozone exposure time	0 min	66.42 ± 4.77 b	13.45 ± 3.92 a	53.74 ± 4.71 a
	15 min	65.71 ± 6.01 a	14.08 ± 3.96 a	53.15 ± 6.20 a
	30 min	65.70 ± 4.81 a	13.76 ± 3.83 a	53.75 ± 5.00 a
Duration of storage	1st day	67.69 ± 4.17 b	12.55 ± 3.99 a	54.95 ± 3.79 b
	30th day	65.54 ± 4.36 a	13.75 ± 3.71 b	53.44 ± 4.41 a
	60th day	64.59 ± 6.39 a	14.99 ± 3.63 c	52.23 ± 6.95 a
	Mean	65.94 ± 5.22	13.76 ± 3.90	53.550 ± 5.33

The data are expressed as mean values (n = 10) ± SD; SD—standard deviation. Mean values within columns with different letters are significantly different (*p* < 0.05).

**Table 4:** The pH and titratable acidity [g·100 g^−1^] of fruits of tested *Chaenomeles japonica* clones. Mean values within columns with different letters are significantly different (*p* < 0.05).

Variables		pH	Titratable Acidity [g·100 g^−1^]
Cultivar	3b/1	2.85 ± 0.19 a	3.39 ± 0.24 b
	4c/1	3.1 ± 0.08 f	3.4 ± 0.21 b
	7c/10	2.88 ± 0.11 b	3.68 ± 0.11 c
	7d/8	3.07 ± 0.12 e	3.23 ± 0.15 a
	8c/12	3.11 ± 0.07 f	3.65 ± 0.18 c
	10d/8	2.91 ± 0.13 c	3.67 ± 0.12 c
	12b/5	3.01 ± 0.10 d	4.04 ± 0.11 d
Ozone exposure time	0 min	2.99 ± 0.19 b	3.57 ± 0.11 a
	15 min	2.97 ± 0.17 a	3.6 ± 0.18 b
	30 min	3.01 ± 0.18 b	3.57 ± 0.14 b
Duration of storage	1st day	2.98 ± 0.11 a	3.64 ± 0.16 b
	30th day	2.99 ± 0.08 a	3.54 ± 0.17 a
	60th day	2.99 ± 0.05 a	3.54 ± 0.18 b
Mean		2.99 ± 0.12	3.58 ± 0.16

Mean values (n = 10) are shown with standard deviation (SD). Significant differences between values in the same column are indicated by different letters (*p* < 0.05).

**Table 5:** Effect of genotype, storage period and ozone treatment time on the chemical properties of *Chaenomeles japonica* fruits.

Variables		AA [mg·100 g^−1^]	TPC [mg GAE·100 g^−1^]	DPPH [mM TE·100 g^−1^]	ABTS [mM TE·100 g^−1^]	FRAP [mM Fe·100 g^−1^]
Breeding clone	3b/1	114.7 ± 17.6 b	373.92 ± 55.36 de	2.37 ± 0.13 ab	1.59 ± 0.10 ab	0.33 ± 0.02 c
	4c/1	117.3 ± 15.7 b	359.14 ± 26.56 bc	2.34 ± 0.13 a	1.64 ± 0.09 c	0.32 ± 0.02 b
	7c/10	115.7 ± 18.1 b	365.11 ± 29.31 cd	2.33 ± 0.14 a	1.56 ± 0.09 a	0.33 ± 0.02 bc
	7d/8	107.2 ± 18.3 a	373.41 ± 27.63 de	2.33 ± 0.13 a	1.64 ± 0.10 c	0.32 ± 0.02 b
	8c/12	108.2 ± 18.5 ab	347.09 ± 25.54 ab	2.33 ± 0.14 a	1.62 ± 0.10 bc	0.32 ± 0.02 b
	10d/8	105.7 ± 17.3 a	361.13 ± 26.70 c	2.40 ± 0.14 b	1.58 ± 0.10 a	0.32 ± 0.02 b
	12b/5	111.7 ± 18.4 ab	343.84 ± 23.85 a	2.38 ± 0.13 ab	1.64 ± 0.09 c	0.31 ± 0.02 a
Ozone exposure time	0 min	107.2 ± 18.4 a	347.78 ± 33.85 a	2.33 ± 0.14 a	1.60 ± 0.10 a	0.32 ± 0.02 a
	15 min	113.2 ± 18.2 b	367.32 ± 29.93 b	2.37 ± 0.13 b	1.62 ± 0.10 b	0.32 ± 0.02 a
	30 min	114.0 ± 17.0 b	366.47 ± 34.30 b	2.35 ± 0.14 ab	1.61 ± 0.10 ab	0.32 ± 0.02 a
Duration of storage	1st day	127.4 ± 7.9 c	396.95 ± 29.37 c	2.56 ± 0.05 c	1.73 ± 0.04 c	0.35 ± 0.01 c
	30th day	116.9 ± 8.3 b	348.05 ± 13.19 b	2.27 ± 0.04 b	1.55 ± 0.04 b	0.31 ± 0.01 b
	60th day	90.1 ± 10.4 a	336.56 ± 18.82 a	2.24 ± 0.04 a	1.53 ± 0.05 a	0.30 ± 0.01 a
Mean		111.5 ± 18.1	360.52 ± 33.89	2.35 ± 0.14	1.61 ± 0.10	0.32 ± 0.02

Mean values (n = 10) are shown with standard deviation (SD). Significant differences between values in the same column are indicated by different letters (*p* < 0.05); AA—Ascorbic Acid, TPC—Total phenolic content.

## Data Availability

The original contributions presented in the study are included in the article, further inquiries can be directed to the corresponding author.
